# Metagenome-Assembled Genomes Reveal Mechanisms of Carbohydrate and Nitrogen Metabolism of Schistosomiasis-Transmitting Vector *Biomphalaria Glabrata*

**DOI:** 10.1128/spectrum.01843-21

**Published:** 2022-03-07

**Authors:** Shuling Du, Xi Sun, Jingxiang Zhang, Datao Lin, Runzhi Chen, Ying Cui, Suoyu Xiang, Zhongdao Wu, Tao Ding

**Affiliations:** a Department of Immunology, Zhongshan School of Medicine, Sun Yat-sen University, Guangzhou, China; b Key Laboratory of Tropical Disease Control, Ministry of Education, Sun Yat-sen University, Guangzhou, China; c Department of Parasitology, Zhongshan School of Medicine, Sun Yat-sen University, Guangzhou, China; d Provincial Engineering Technology Research Center for Biological Vector Control, Guangzhou, China; Tufts University

**Keywords:** metagenome-assembled genomes, gut microbiota, *Biomphalaria glabrata*, *Schistosoma mansoni*, metabolism

## Abstract

*Biomphalaria glabrata* transmits schistosomiasis mansoni which poses considerable risks to hundreds of thousands of people worldwide, and is widely used as a model organism for studies on the snail-schistosome relationship. Gut microbiota plays important roles in multiple aspects of host including development, metabolism, immunity, and even behavior; however, detailed information on the complete diversity and functional profiles of *B. glabrata* gut microbiota is still limited. This study is the first to reveal the gut microbiome of *B. glabrata* based on metagenome-assembled genome (MAG). A total of 28 gut samples spanning diet and age were sequenced and 84 individual microbial genomes with ≥ 70% completeness and ≤ 5% contamination were constructed. *Bacteroidota* and *Proteobacteria* were the dominant bacteria in the freshwater snail, unlike terrestrial organisms harboring many species of *Firmicutes* and *Bacteroidota*. The microbial consortia in *B. glabrata* helped in the digestion of complex polysaccharide such as starch, hemicellulose, and chitin for energy supply, and protected the snail from food poisoning and nitrate toxicity. Both microbial community and metabolism of *B. glabrata* were significantly altered by diet. The polysaccharide-degrading bacterium *Chryseobacterium* was enriched in the gut of snails fed with high-digestibility protein and high polysaccharide diet (HPHP). Notably, *B. glabrata* as a mobile repository can escalate biosafety issues regarding transmission of various pathogens such as Acinetobacter nosocomialis and Vibrio parahaemolyticus as well as multiple antibiotic resistance genes in the environment and to other organisms.

**IMPORTANCE** The spread of aquatic gastropod *Biomphalaria glabrata*, an intermediate host of Schistosoma mansoni, exacerbates the burden of schistosomiasis disease worldwide. This study provides insights into the importance of microbiome for basic biological activities of freshwater snails, and offers a valuable microbial genome resource to fill the gap in the analysis of the snail-microbiota-parasite relationship. The results of this study clarified the reasons for the high adaptability of *B. glabrata* to diverse environments, and further illustrated the role of *B. glabrat*a in accumulation of antibiotic resistance in the environment and spread of various pathogens. These findings have important implications for further exploration of the control of snail dissemination and schistosomiasis from a microbial perspective.

## INTRODUCTION

The spread of aquatic gastropod *Biomphalaria glabrata*, an intermediate host of Schistosoma mansoni (1), exacerbates the burden of schistosomiasis disease in sub-Saharan Africa, the Middle East, and South America (2). In addition, the snail also supports the life cycle of a variety of nematode parasites including *Angiostrongylus cantonensis*, the causative agent of human eosinophilic meningitis (3). *B. glabrata* lives in ponds, lakes, streams, and irrigation channels, and mainly feeds on decaying plant matter. As the widespread geographic distribution of *B. glabrata* is closely related to its feeding habits, investigation of the correlation between freshwater snail and its eating pattern is beneficial to address the ecological questions of invasive species. Gut microbiota is well known to play a crucial role in host nutrition digestion, xenobiotic metabolism, immunity, environmental adaptability, and even behaviors. Therefore, research on snails, especially their adaptability and livability, from the perspective of gut microbiota has received increasing attention. Scientists now have a preliminary understanding of snail gut microbiota composition by using high-throughput culture method or 16S rRNA gene sequencing (4–7). However, culturomics techniques cannot identify the so-called “not yet culturable” microorganisms and 16S rRNA sequencing is limited by the heterogeneity of amplification and low resolution of species annotation (8). Owing to these serious shortcomings, complete bacterial diversity and functional traits of *B. glabrata* have remained uncharacterized.

Metagenomics sequencing and metagenome-assembled genome (MAG) provide alternative methods for identifying uncultivated microbiota and obtaining functional information of specific microbiota, and have been extensively applied to human, pig, and mouse microbiome studies (9–11). MAGs are potentially useful for obtaining whole-genome sequences of unculturable microorganisms, discovering new species and analyzing their functions, and contributing to the study of the composition and function of microbiota of “interest.”

In the present study, we applied shotgun sequencing to 28 gut samples of *B. glabrata* and constructed 84 microbial genomes by leveraging assembly and binning without any reference genomes to (i) characterize the taxonomic composition of snail intestine microbiota and quantify their abundance, (ii) understand the metabolic capacity, particularly carbohydrate degradation and nitrogen utilization, (iii) assess the influence of diet on bacterial diversity and functions, and (iv) estimate the potential risk of transmission of pathogens and antibiotic resistance genes (ARGs).

By utilizing metagenome sequencing, assembly, and binning, this study is the first to assemble the microbial genomes, which can be used for future research, as well as reveal the gut bacterial composition and basic functions of *B. glabrata.* Besides, the importance of microbial carbohydrate and nitrogen metabolism in the invasion and spread of *B. glabrata* was also analyzed. The results of this study enhance our understanding of the biological characteristics of *B. glabrata*, advocate gut microorganisms as potential targets for interfering with the physiological activities of the host snail, and provide new insights for future prevention and control of snail dissemination and even schistosomiasis.

## RESULTS

### Description of study design.

**FIG 1:**
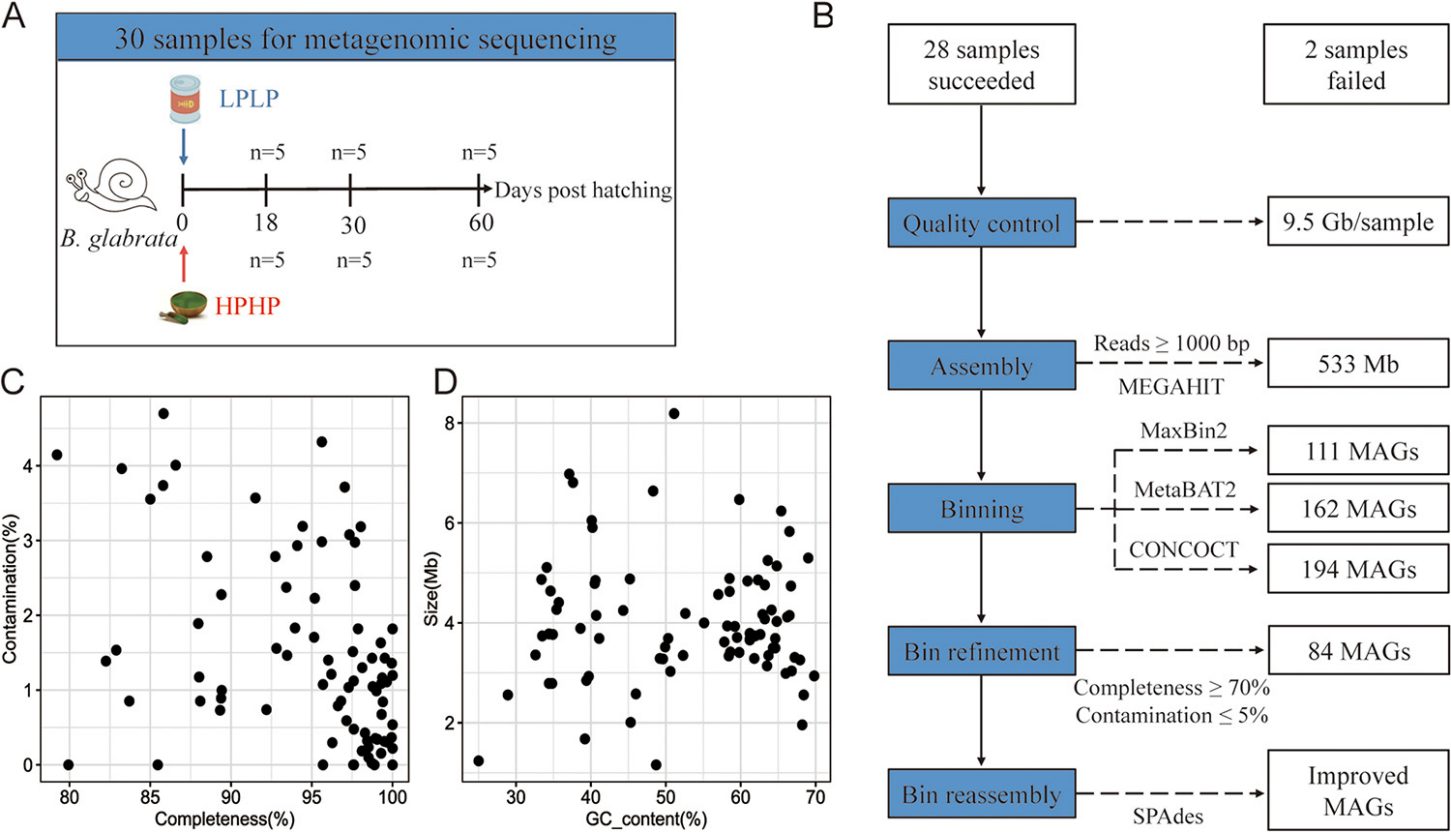
Generation of metagenome-assembled genomes (MAGs) of *B. glabrata*. (A) Study design. After hatching, the snails were fed with two types of diets, low-digestibility protein and low polysaccharide diet (LPLP) and high-digestibility protein and high polysaccharide diet (HPHP). A total of 30 gut tissues were collected at three different time points (days 18, 30, and 60) for sequencing. (B) Flowchart of the procedure for metagenomic analysis. Blue boxes indicate the pipeline for building MAGs, including quality control, assembly, and binning, and white boxes show the outcomes of each step. (C) Completeness and contamination rate of individual MAG. One point represents one MAG. (D) GC content and genome size of individual MAG.

To collect gut microorganisms from *B. glabrata* in a variety of physiological states and to construct a broadly representative metagenome, we included two important variables that may affect gut microbial composition, diet, and age. The hatched snails were fed with two experimental diets, low-digestibility protein and low polysaccharide diet (LPLP) and high-digestibility protein and high polysaccharide diet (HPHP), for 60 days, both of which had been reportedly used to rear snails in the laboratory but with different nutrition elements (12, 13). Five snails from each group were sampled on days 18, 30, and 60, representing three stages of snail development, namely, juvenile, youth, and adult (Fig. 1A). A total of 30 *B. glabrata* snails were collected for shotgun metagenomic sequencing. Owing to poor integrity or purity of the extracted genomic DNA, two samples from HPHP group failed to produce sequencing libraries; thus, 28 metagenomes were completed for subsequent analysis (Table S1). After pre-processing of the sequencing data, 266 Gb of clean paired-end data were obtained, with an average sequencing depth of 9.5 Gb per sample. Given the limited sample size and sequencing depth, we used MEGAHIT, which assembled clean sequences from 28 samples pooled together, with the aim of using merged assembly instead of a single-sample assembly strategy to generate long and complex overlapping contigs. As a result, a total of 533 Mb of sequence containing 115,151 contigs of ≥ 1,000 bp was generated, with an N50 of 12,658 bp (Table S2).

### Novel reference with 84 MAGs was constructed for *B. glabrata*.

The overall bioinformatic workflow of binning is shown in Fig. 1B. By using the contigs and quality-controlled sequences, binning was performed without any reference genomes. Three mainstream binning algorithms, MaxBin2, MetaBAT2, and CONCOCT, were used in parallel, which produced 111, 162, and 194 MAGs, respectively. As these three binning methods may yield duplicate draft genomes and complement each other, a set of MAGs consisting of these 467 MAGs were de-replicated and refined with the MetaWRAP-Bin_refinement module. Finally, 84 unique MAGs complied with the threshold requirements of ≥ 70% completeness and ≤ 5% contamination. To improve the quality, the obtained putative draft genomes were further reassembled with SPAdes, and the original bins were replaced when the reassembled ones exhibited better quality in terms of completeness and contamination. A total of 46 original MAGs were improved. Overall, the completeness of the 84 MAGs ranged from 79.22% to 100% (Fig. 1C). Among these, 77.4% (65/84) of the MAGs satisfied high-quality criteria (> 90% completeness and < 5% contamination) (14), the genomic size was 1.2 to 8.2 Mb, and the GC content was 25% to 70% (Fig. 1D, Table S3).

To estimate the representation of these MAGs and investigate the bacterial diversity of *B. glabrata*, the relative abundance of MAGs was calculated by aligning clean reads to reference sequences (Table S4). The 84 MAGs were noted to recall 90.18% of clean paired-end data, far exceeding that reported in Tibetan Pig (38%) (15). When the filtering threshold (≥ 50% completeness and ≤ 10% contamination) was lowered to recover more microbial genomes, the recall rate (90.41%) was not increased, although 105 MAGs were obtained. This result suggested that further improvement in read mapping at the expense of quality was difficult for this data set; therefore, the 84 MAGs obtained were inferred as adequate for subsequent analysis.

### *B. glabrata* has the potential to transmit putative pathogens and ARGs.

**FIG 2:**
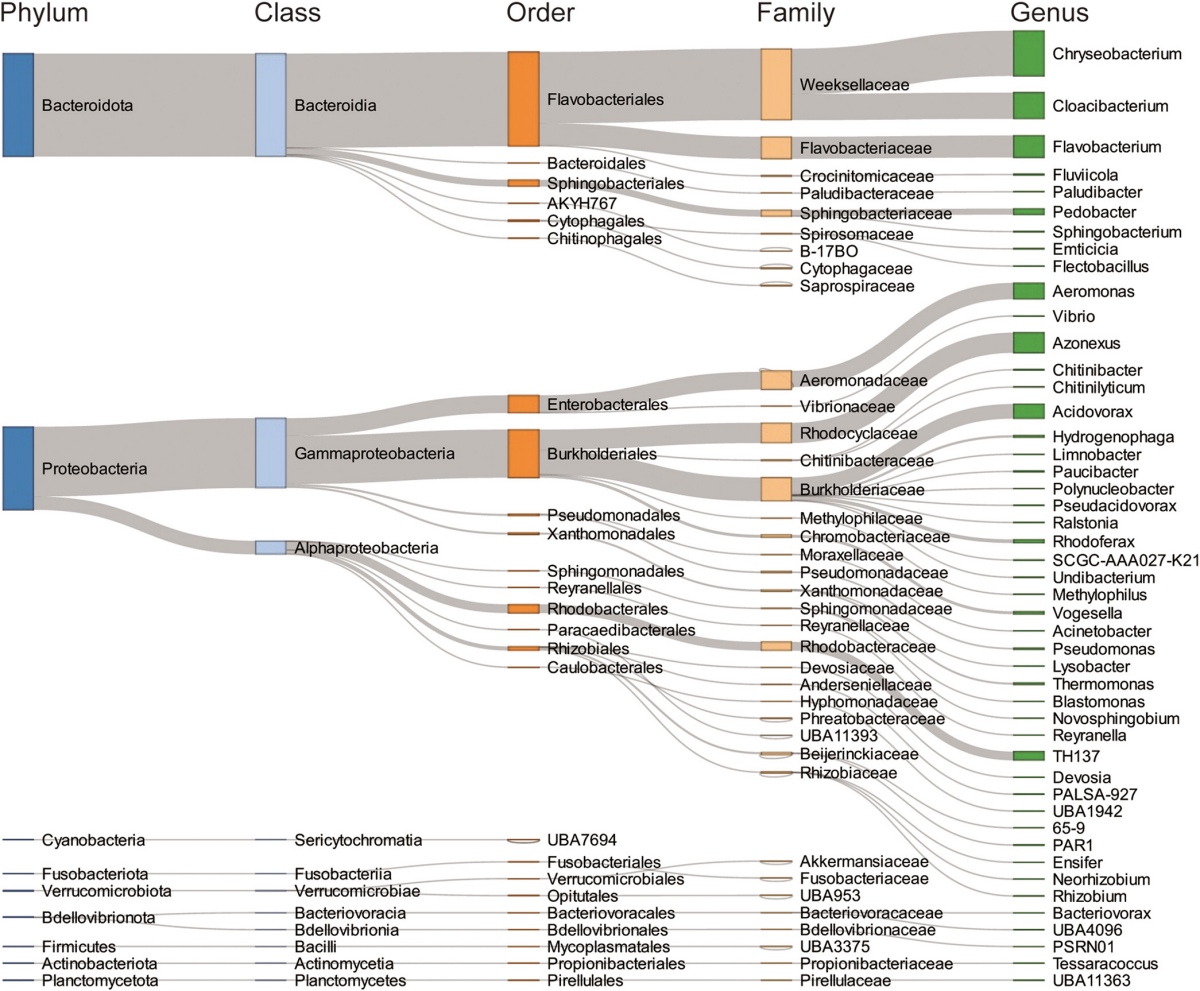
Metagenome-assembled genomes (MAGs) taxonomy across different levels. Sankey plot depicts the detailed classifications and proportions of MAGs at five phylogenetic levels (phylum, class, order, family, and genus). Height of the block represents microbial abundance.

All 84 MAGs could be annotated to the family level and belonged to one kingdom, nine phyla, 11 classes, 25 orders, and 38 families. A total of 65 MAGs were assigned to 60 genera (Table 1), and the detailed classification results are shown in Table S3. When compared with terrestrial animals such as pigs, murine, and termites (Table 2) (9–11), *B. glabrata* harbored relatively simple microbial consortia in the laboratory. While the gut of terrestrial organisms is known to contain many species of *Firmicutes* and *Bacteroidota*, the vast majority of MAGs in *B. glabrata* can be attributed to *Proteobacteria* (54/84) and *Bacteroidota* (19/84), similar to that of another freshwater animal, zebrafish (16). Fig. 2 illustrates the microbial composition and average abundance in *B. glabrata* snails at five different phylogenetic levels. *Bacteroidota* (49.41%) was the most dominant phylum, followed by *Proteobacteria* (39.80%). The top 10 microbial genera (79.21%) were *Chryseobacterium* (21.64%), *Cloacibacterium* (12.57%), *Flavobacterium* (10.54%), *Azonexus* (9.62%), *Aeromonas* (7.81%), *Acidovorax* (6.98%), *TH137* (4.13%), *Pedobacter* (2.89%), *Rhodoferax* (1.71%), and *Vogesella* (1.32%). Most of these genera have also been detected in other snails in previous studies based on 16S rRNA sequencing (7, 17–20), and among them, *Chryseobacterium*, *Cloacibacterium*, and *Flavobacterium* are affiliated with the aerobic order *Flavobacteriales*, which are well-known for lipid and carbohydrates metabolism (21, 22), while members of *Burkholderiales*, including *Azonexus*, *Acidovorax*, *Rhodoferax*, and *Vogesella*, are hydrocarbon-utilizing and denitrifying bacteria (23, 24). These results suggested that *B. glabrata* microbiome may play an important role in host metabolism and physiological adaptations, prompting further exploration of carbon and nitrogen metabolic pathways.

**TABLE 1:** Taxonomic classification of 84 MAGs at different levels

Levels	MAG	Unique taxa	Relative abundance of major microbes (%)
Kingdom	84	1	*Bacteria* (90.18)
Phylum	84	9	*Bacteroidota* (49.41), *Proteobacteria* (39.80)
Class	84	11	*Bacteroidia* (49.41), *Gammaproteobacteria* (33.37), *Alphaproteobacteria* (6.43)
Order	84	25	*Flavobacteriales* (45.15), *Burkholderiales* (23.06), *Enterobacterales* (8.45), *Rhodobacterales* (4.13)
Family	84	38	*Weeksellaceae* (34.21), *Burkholderiaceae* (11.29), *Flavobacteriaceae* (10.54), *Rhodocyclaceae* (9.62), *Aeromonadaceae* (8.35), *Rhodobacteraceae* (4.13)
Genus	65	60	*Chryseobacterium* (21.64), *Cloacibacterium* (12.57), *Flavobacterium* (10.54), *Azonexus* (9.62), *Aeromonas* (7.81), *Acidovorax* (6.98), *TH137* (4.13)
Species	14	14	Aeromonas jandaei (6.00), *Acidovorax_D temperans* (4.14), *Flavobacterium sp004119495* (2.97), Aeromonas allosaccharophila (2.21), *Acidovorax_D sp000302535* (2.14)

**TABLE 2:** Comparison of taxonomic results of *B. glabrata*, Danio rerio, termite, mouse, and pig

Phylum	*B. glabrata* (%)	Danio rerio (%)	Termite (%)	Mouse (%)	Pig (%)
*Proteobacteria*	54 (64.3)	71 (60.7)	67 (12.2)	11 (1.2)	167 (2.7)
*Bacteroidota*	19 (22.6)	18 (15.4)	33 (6.0)	123 (13.5)	1,061 (16.9)
*Firmicutes*	1 (1.2)	4 (3.4)	237 (43.0)	707 (77.5)	3,989 (63.5)
*Actinobacteriota*	1 (1.2)	9 (7.7)	71 (12.9)	23 (2.5)	262 (4.2)
*Verrucomicrobiota*	3 (3.6)	1 (0.9)		2 (0.2)	149 (2.4)
*Planctomycetota*	1 (1.2)		12 (2.2)		17 (0.3)
*Fusobacteriota*	1 (1.2)	1 (0.9)			5 (0.1)
*Cyanobacteria*	1 (1.2)			8 (0.9)	161 (2.6)
Others	3 (3.6)	13 (11.1)	131 (23.8)	38 (4.2)	474 (7.5)
Total	84	117	551	912	6,285

A total of 14 MAGs could be annotated to species-level taxonomy. Among them, four microbial species in *B. glabrata* were opportunistic pathogens, with members of Aeromonas jandaei (MAG.60), a common pathogen in fish and immunocompromised patients (25), accounting for 5.60%, followed by members of Ralstonia pickettii (MAG.12, 0.34%), Acinetobacter nosocomialis (MAG.29, 0.21%), and Vibrio parahaemolyticus (MAG.51, 0.10%), all of which cause bacterial infections in humans (26–28). These findings indicated that snails not only transmit parasites, but can also spread potentially pathogenic bacteria that can damage human or aquatic organisms health.

**FIG 3:**
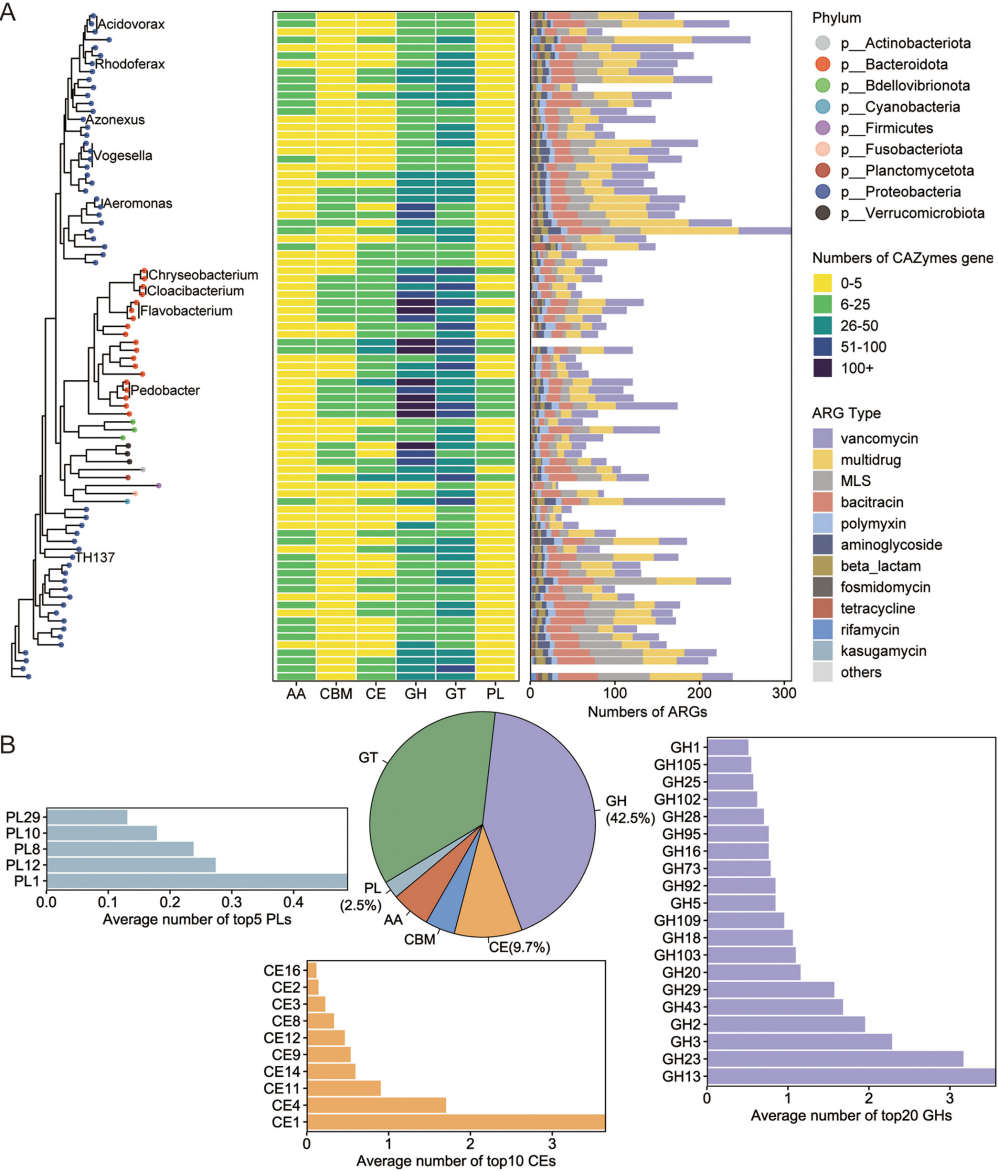
Phylogeny and distribution of carbohydrate-active enzymes (CAZymes) and antibiotic resistance genes (ARGs) types of the recovered metagenome-assembled genomes (MAGs). (A) Phylogenetic relationships and numbers of genes associated with CAZymes and antibiotic resistance among 84 MAGs. Left: Phylogenetic tree of all the resolved taxonomies. Points with different colors represent different phyla as indicated by the color code (top right); clades of the top 10 abundant genera are annotated with text label. Middle: Heatmap displaying counts of genes belonging to each CAZymes family in each MAG. Color gradient (middle right) represents the numbers of CAZymes genes. AA, auxiliary activity; CBM, carbohydrate-binding module; CE, carbohydrate esterase; GH, glycoside hydrolase; GT, glycosyltransferase; PL, polysaccharide lyase. Right: Stacking histogram displaying the numbers of ARGs in each MAG. Colors (lower right) represent the ARG type; the five ARG types with the lowest number are merged as a whole and defined as “others.” MLS, macrolide-lincosamide-streptogramin. (B) Information on the target three CAZymes families. Pie chart displaying proportions of six CAZymes families. Bar chart showing the average numbers of top 20 GHs, top 10 CEs, and top 5 PLs families in each MAG.

As the spread of ARGs and antibiotic-resistant bacteria has become an urgent health issue in the age of urbanization and industrialization (29, 30), the present study also evaluated the risk of host snail as a silent and mobile ARGs reservoir. Fig. 3A provides information on each MAG carrying ARGs. Except for MAG.44 and MAG.64, in which no ARGs were detected, the other 82 MAGs were found to carry 86 ARG subtypes belonging to 16 ARG types (Table S5). The five most frequently detected ARG types were vancomycin, multidrug, macrolide_lincosamide_streptogramin, bacitracin, and polymyxin. These predominant ARG types were observed to be similar to those previously reported for a large drinking water reservoir (31). In the present study, an average of 133 ARGs were detected among the 82 MAGs, which is significantly higher than those observed in MAGs from sludge reactors (3 ARGs) (32). The members of *Proteobacteria* (153.49) harbored more ARGs than those of *Bacteroidota* (93.89) (Table S5), which is consistent with the fact that *Proteobacteria* are considered as the potentially dangerous repository of ARGs across diverse environments, because they can uptake foreign free DNA through natural transformation and transmit these ARGs to other ecosystems (33).

### Gut microbiome contributes to polysaccharide and nitrite metabolism in snails.

The characterization of gut microbiota of *B. glabrata* can also provide data on microbial function, which can help to understand the metabolic functions of snail and its ability to adapt to a wide range of environments. In the present study, 7,730 carbohydrate-active enzyme (CAZyme) genes in 84 MAGs were characterized. The highest number of genes encoded glycoside hydrolases (GHs, 3,287), followed by glycosyltransferases (GTs, 2,736), carbohydrate esterases (CEs, 752), auxiliary activities (AAs, 431), carbohydrate-binding modules (CBMs, 330), and polysaccharide lyases (PLs, 194) (Fig. 3B). When compared with species belonging to *Proteobacteria* among the top 10 genera, *Chryseobacterium* (MAG.19 133; MAG.63 136), *Cloacibacterium* (MAG.2 110; MAG.11 180), and *Flavobacterium* (MAG.42 234; MAG.53 146; MAG.75 200) had higher abundance of CAZyme gene (Fig. 3A), suggesting that these bacteria gained the ability to efficiently use carbon sources. This finding also explained the reason for the proportion of *Flavobacteriales* (44.75%) in the gut microbiota of *B. glabrata*.

To investigate the carbohydrate-digestive capacities in snail guts, the families of GHs, CEs, and PLs that can directly break down the glycoside bond to convert complex polysaccharide into small oligosaccharide were analyzed (Fig. 3C). The most abundant GH gene in the snail gut was GH13, which encodes an alpha-amylase for starch degradation. In addition, GH3, GH43, and GH39, which encode β-xylosidase, a hemicellulolytic enzyme that hydrolyzes xylooligosaccharides to xylose and thus digest polysaccharide xylan from plant cell wall, were also found in abundance. CE1 and CE4 members, which catalyze de-acylation of xylan, are vital accelerators for xylan degradation. Besides, enzymes that can degrade pectin, including GH16, GH28, and PL1, were also identified. When compared with hemicellulases-encoding genes, the abundance of cellulose-encoding gene (GH5) was low. In addition, certain number of GH20, GH18, and GH92 genes, which encode enzymes capable of decomposing chitin that makes up the exoskeleton of crustaceans and shells of mollusks, was also detected in the snail. Owing to the metabolic capacity of these microbial enzymes, the omnivorous snail *B. glabrata*, with a wide range of feeding habits, could extensively use bacterial biofilms, algae, diatoms, decaying macrophytes, and carrion as a source of nutrition, and this ability could be one of the main factors that enables snails to invade, colonize, and spread to new areas.

**FIG 4:**
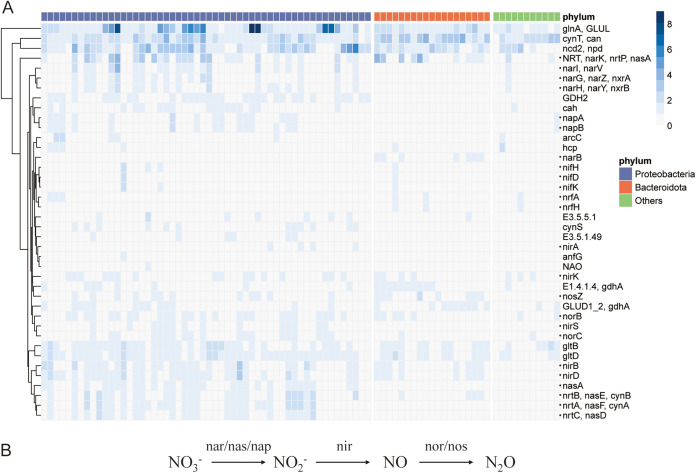
Nitrogen metabolic pathway of the recovered metagenome-assembled genomes (MAGs). (A) Distribution of nitrogen metabolism associated gene counts in each MAG. Color gradients represent the counts. Black dots mark the denitrifying genes. (B) Schematic representation of the major denitrifying pathways.

The results of taxonomic annotation also suggested the presence of many *Proteobacteria*, including *Azonexus*, *Acidovorax*, *Rhodoferax*, and *Vogesella*, from known groups containing denitrifying bacteria. Denitrification, a pathway for permanently removing nitrogen in fluvial network, is the process of converting nitrate (NO_3_-) to nitrous oxide (N_2_O) and molecular nitrogen gas (N_2_) through a series of microbial activities (34). Many aquatic organisms have been identified to emit N_2_O in NO_3_--rich ecosystems such as freshwater (35). To confirm the presence of denitrification-associated metabolic pathways in snails, we aligned 84 MAGs to the KEGG database for functional annotation. Interestingly, a large number of nitrogen metabolism genes were enriched in the denitrification process, especially in *Proteobacteria* (Fig. 4A). The enzymes catalyzing the reactions include nitrate reductase (nar/nas/nap) reducing NO_3_- to nitrite, nitrite reductase (nir) reducing nitrite to nitric oxide, and nitric oxide reductase (nor) reducing nitric oxide to N_2_O (Fig. 4B). Nitronate monooxygenase (ncd2/npd) identified in snail has been shown to oxidize mitochondrial toxic metabolite, propionate 3-nitronate, in fungi, plants, and animals (36), suggesting that the gut bacteria of *B. glabrata* may perform a detoxification mechanism or remove nitrogen from the living environment. In addition, a certain amount of glutamate synthase (glt) along with glutamine synthase (gln), which can regulate nitrogen assimilation (37), was also detected. Under the conditions of nitrogen starvation, glutamate synthase and glutamine synthase are activated to synthesize nitrogen-containing nutrients, suggesting that the gut microbiota of snails can prevent the host snails from nitrite and food poisoning, as well as synthesize essential nitrogenous components to cope with nutrition limitation.

### Diet and age affect the gut bacterial composition and function of snails.

**FIG 5:**
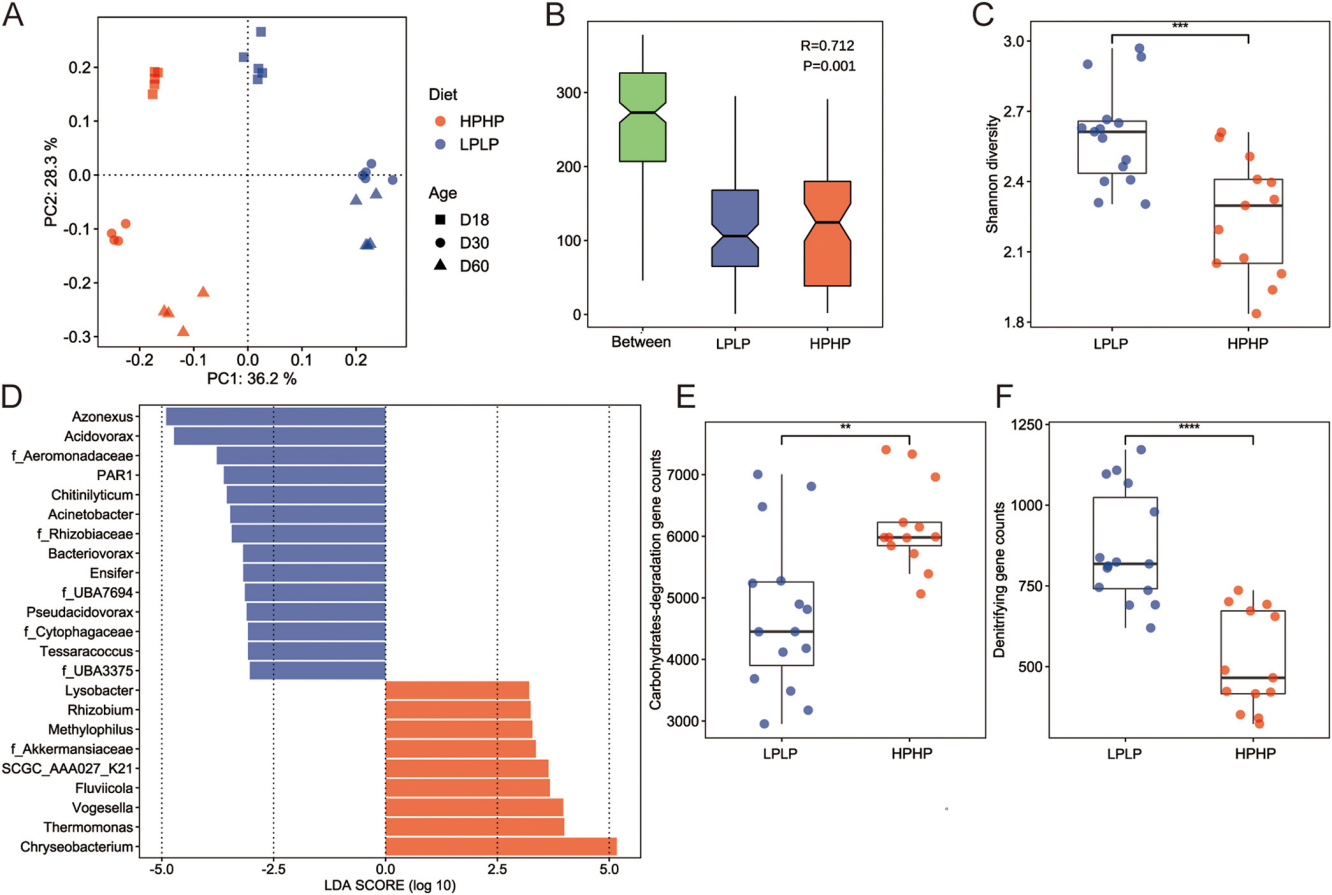
Effect of diets and ages on *B. glabrata* gut bacterial community. (A) Bray-Curtis dissimilarity of the gut microbial community of *B. glabrata* fed with different diets at three time points and visualized as PCoA plot. (B) Analysis of similarity using Bray-Curtis dissimilarity indices assessing the difference between and within groups. “Between” represents the mean of ranked dissimilarities between low-digestibility protein and low polysaccharide diet (LPLP) and high-digestibility protein and high polysaccharide diet (HPHP) groups. (C) Shannon diversity of the gut microbiota of snails fed with different diets. Statistical analysis was performed using Wilcoxon test. (D) LEfSe analysis (LDA significant threshold (log_2_)>±3.0) identified the discriminative genera between the two diet groups. Blue bars denote bacteria enriched in the LPLP group and red bars indicate bacteria enriched in the HPHP group. (E) Comparison of total carbohydrate-degrading genes counts, including glycoside hydrolases, carbohydrate esterases, and polysaccharide lyases between the two groups. Statistical analysis was performed using Wilcoxon test. (F) Comparison of the total denitrifying genes counts between the two diet groups. Statistical analysis was performed using Wilcoxon test.

To assess the effects of diet and age on the microbial community of *B. glabrata*, principal coordinates analysis (PCoA) based on Bray-Curtis dissimilarity was used, and significant differences (Fig. 5A, PERMANOVA, *P < *0.05) were detected. The results of analysis of similarities (ANOSIM) showed lower intragroup variation than intergroup variation (Fig. 5B), which supported the rationality of diet-based grouping despite the presented confounding factor of age. Snails fed with LPLP diet exhibited higher Shannon diversity than those on HPHP diet, which might be owing to the fact that HPHP is a single-component spirulina powder, while LPLP is a mixture of multiple components (Fig. 5C). Furthermore, LEfSe analysis was applied to identify microbial biomarkers between the two groups (Fig. 5D), and *Chryseobacterium* was found to be enriched in snails fed with HPHP diet, whereas *Acidovorax* and *Azonexus* were significantly elevated in snails fed with LPLP diet. Diet not only altered the microbiome community structure in snail, but also influenced the functional pathways. The functional annotation data showed that the HPHP group had a higher number of carbohydrate-degrading genes, whereas the LPLP group had a higher number of denitrifying genes (Fig. 5E and F). This finding indicated that a high polysaccharide diet stimulates the snail gut microbiota to secrete more CAZymes, whereas a low-digestible protein diet requires higher NO_3_- content in the aquaculture system and more denitrifying enzymes. Time-series analysis across the three time points revealed that *Acidovorax*, *Azonexus*, and *Chryseobacterium* exhibited similar trends in the two diet groups over time (Fig. S1A–F). Comparison of the metabolic capacities of snails after 30 and 60 days post-hatching demonstrated an increase in the number of carbohydrate-degrading genes and a decrease in denitrifying genes (Fig. S1G–J). These results illustrated that diet and age are significantly involved in shaping specific microbial communities in *B. glabrata* and influencing the metabolic capacity of the host.

## DISCUSSION

The present study is the first to implement shotgun metagenomic sequencing and assembly for the analysis of gut microbiota of schistosomiasis-transmitting vector, *B. glabrata*. A total of 84 MAGs were constructed, which can offer an important reference for future studies on the microbial structure and functional profiles of freshwater snails. According to the MAGs data set, the intestinal microbiota of *B. glabrata* was noted to harbor a large quantity of genes encoding carbohydrate-degrading enzymes (such as amylase, hemicellulose, and chitinase) for energy supply and nitrogen transformation related enzymes for detoxification. Besides, the present study also demonstrated that diet and age influenced the development of diverse microbial communities.

*B. glabrata* is a major vector for the transmission of hepatosplenic schistosomiasis. As there is still no vaccine available against *Schistosoma*, intervening the emergence and spread of host snails is still the most important strategy for eliminating the prevalence of schistosomiasis. Although the only drug recommended by the World Health Organization available for large-scale field use against schistosomiasis transmission is the molluscicide niclosamide, this drug has caused severe drug resistance and threatened other aquatic animals, prompting for the development of new technologies to reduce the risk of schistosomiasis spread in field. Gut microbiota is critical to animal health, and has been considered in recent years as a potential target for preventing vector-borne diseases (e.g., *Wolbachia*-based approaches for arboviruses control) (38). The gut microbiota of aquatic invertebrates is still less explored and warrants further research to characterize the symbiotic relationships between host and indigenous microorganisms. A comprehensive knowledge of the compositional and functional baseline of the snail microbiome can support our understanding of the mechanism underlying the high adaptability and widespread invasion of snails.

When compared with hundreds of MAGs isolated from terrestrial organisms, the microbial diversity of laboratory-reared freshwater snail was observed to be relatively low. *B. glabrata* gut microbiota was dominated by *Bacteroidota* and *Proteobacteria*, and its top 10 microbial genera accounted for 79.21% of the gut microbial population, among which many bacteria were common in both aquatic gastropods and natural water environment (18–20, 39). In particular, *Chryseobacterium*, *Cloacibacterium*, and *Flavobacterium*, which were abundant (> 10%) in *B. glabrata*, have also been characterized in the gut microbiota of other snails and freshwater sediments (18, 20, 40). Freshwater snails cannot survive out of water and their microbiota may vary depending on their habitats. Therefore, wild-reared snail microbiome requires further investigations, and future studies should develop an experimental design across age, geography, lifestyle, and pathogen infection to reconstruct more MAGs to increase the coverage.

Bacteria with the potential to degrade plant components are common in the gut of snails (41). In the present study, functional analysis revealed the enrichment of a number of microbial genes in *B. glabrata* gut, which help in the digestion of starch (GH13), hemicellulose (GH3, GH43, GH39, CE1, CE4), and chitin (GH20, GH18, GH92) commonly found in algae, diatoms, decaying macrophytes, fungi, insects, and crustaceans. The carbohydrate-degrading enzymes found in *B. glabrata* gut were noted to be similar to those in slug and giant snail (42). The enzymes detected in the gut microbiota of *B. glabrata* may have potential biotechnological applications in a variety of industrial processes. Interestingly, the proportion of cellulose-degrading enzymes in *B. glabrata* digestive system was lower than that of hemicellulose-degrading enzymes, which was consistent in slug and giant snail. To absorb more substances and acquire sufficient energy as efficiently as possible, the mollusk microbiota might prefer to utilize easily digestible polysaccharide rather than recalcitrant cellulose. In addition to carbohydrate metabolism, the results of the present study showed that the gut microbiota of *B. glabrata* has significant potential to metabolize NO_3_-. High concentrations of NO_3_- in freshwater ecosystems affect the health of aquatic organisms by forming pigments that are unable to carry oxygen (43). Filter- and deposit-feeding organisms have been reported to convert NO_3_- in the living environment into N_2_O, which might be owing to the presence of denitrifying bacteria in the gut (35). In the present study, the impact of mollusks microbiome on nitrogen cycling was demonstrated and the hypothesis that gut denitrifying bacteria protect the host from NO_3_- toxicity was proposed.

Differences in mollusks phylogeny, habitat, physiology, defense system, and food sources are known to contribute to the variation in the snail gut microbiome (6, 7, 20, 39). In this study, diet and age of *B. glabrata* were found to have significant impacts on the bacterial diversity and metabolism. However, it must be noted that given the limited sample counts, different-age snails fed on same diet were pooled into one group to assess the effect of diet. The ANOSIM results showed that intragroup variation was lower than intergroup variation, and supported the rationality of diet-based grouping despite the confounding factor, age. Subsequently, the effect of age on snail’s gut microbiome was assessed based on the two diets. With the growth of the snail, its intestinal capacity to digest and absorb carbohydrates increased, whereas the ability to detoxify NO_3_- reduced, which may be owing to the development and maturation of snail intestine.

As a necessary component of S. mansoni life cycle, along with advantages such as easy maintenance and manipulation under laboratory conditions, *B. glabrata* has become a common model organism to study snail-*Schistosoma* interactions following successive completion of the whole genome of S. mansoni and *B. glabrata* (44, 45). Initially, we speculated whether snail could be used as a model organism to study the role of gut microbiota in pathogen-host immune interactions (7). Nevertheless, considering other germfree organisms, germfree snails could be a very important resource in which the specificity of a single microorganism toward the host in response to infection by *Schistosoma* or other pathogens could be determined, and our current metagenomics studies on *B. glabrata* gut bacteria can undoubtedly provide the necessary insights. Research on host-microbiota-parasite associations should avoid potential confounding factors because the results of the present study suggested that diet has a strong influence on microbiota composition and function. As different laboratories feed laboratory-reared snails with various diets, including fresh or boiled leaf lettuce, fish food, and spirulina, there is a need to develop a standard breeding protocol.

As a notable invasive species introduced to Egypt from the Caribbean Sea, *B. glabrata* is not only a schistosomiasis-transmitting vector, but also carries the risk of transmission of other infectious pathogens and ARGs. The present study identified four potential bacterial pathogens, namely, *A. jandaei*, *R. pickettii*, A. nosocomialis, and V. parahaemolyticus in *B. glabrata*, which are harmful to aquatic organisms health, economy, food security, and public health. Besides, numerous ARGs were also determined to be enriched in the snail’s microbiome, with the most abundant ARGs being vancomycin resistance genes, which were detected in 82 MAGs. The antibiotic vancomycin is well known as the last line of defense against multiple drug resistant bacteria, including Staphylococcus aureus (46). Thus, these findings suggest the importance of further assessing potential pathogens and ARGs carried by aquatic organisms, especially, the risk of transmission of these potential pathogens and ARGs to other animals or environments. In conclusion, the present study provides important information on gut microbial genome, composition, and function to better understand the physiological properties of the neglected but widely distributed aquatic organism, *B. glabrata*, offering novel insights into parasite-carrying aquatic organisms.

## MATERIALS AND METHODS

### Animal management.

The Puerto Rico strain of *B. glabrata* used in this study was originally obtained from Jiangsu Institute of Parasitic Diseases and maintained at the laboratory of Zhongshan School of Medicine, Sun Yat-sen University, China, for at least 10 generations. The snails were housed in an aquarium tank filled with dechlorinated tap water which was replaced once every 2 days, and maintained at 25°C to 27°C with a photoperiod of 12:12 h (light:dark) (47). *B. glabrata* individuals after hatching were randomly and evenly divided into two groups, and fed with low-digestibility protein and LPLP and HPHP, respectively. The LPLP diet has been fed *B. glabrata* in our laboratory regularly and is a commercial fish diet containing ingredients of animal and plant origin, such as fish meal, corn protein, wheat flour, yeast, and spirulina flour, with about 20% soluble carbohydrates and about 40% hard-to-digest total protein. HPHP diet is pure spirulina powder, which contains 24% carbohydrates and about 60% easily digestible and absorbable proteins.

### Sample collection, DNA extraction, and metagenomic sequencing.

Five snails were collected from each group on days 18, 30, and 60 post-hatching, and their shell surfaces were wiped with 70% ethanol and then rinsed with autoclaved water. All the dissections were performed under sterile conditions to avoid environmental contamination. After careful removal of the shell fragments, the intestines were gently separated with the help of a dissecting microscope, transferred to a 2-mL screw cap tube, and stored at −80°C in a freezer. As the snails collected on day 18 post-hatching were too small to be dissected, the whole body was directly used for subsequent DNA extraction.

DNA was extracted from 30 snail samples, three positive controls, and one negative control (autoclaved water) by using the cetyltrimethylammonium bromide (CTAB) protocol. The three positive controls, comprising two, seven, and 44 human-derived bacteria as mock communities, and one negative control were used to ensure that the sequencing was performed correctly and to provide a level of confidence in the data. Agarose gel electrophoresis and Qubit 3.0 Flurometer (Invitrogen, USA) were used to assess DNA integrity and purity. As the DNA concentration of negative control was too low to prepare the sequencing library, the negative control was excluded from subsequent sequencing. The genomic DNA of the remaining samples was arbitrarily fragmented into 350 bp in size by Covaris Crusher, end-polished, A-tailed, ligated with the adapter, and sequenced on Illumina NovaSeq 6000 platform (provided by Novogene, China).

### Metagenome assembly and binning.

Raw sequences of metagenomics were quality-controlled prior to the assembly, sequencing adapters were filtered, and host contamination was removed. Reads with low quality or mapped to *B. glabrata* genome (44) were filtered out by KneadData (https://huttenhower.sph.harvard.edu/kneaddata/) by setting the options: trimmomatic-options “SLIDINGWINDOW:4:20 MINLEN:50.” Subsequent assembly and binning were processed by MetaWRAP (v1.2.1), a flexible modular pipeline for metagenomic analysis (48). The MetaWRAP-Assembly module completely utilized ultra-fast and memory-efficient assembler MegaHit to undertake assembly with default options (49), and provided reports for users to understand assembly quality. All the assembled contigs of ≥ 1,000 bp were binned with the MetaWRAP-Binning module exploiting three mainstream computational tools: MaxBin2 (50), MetaBAT2 (51), and CONCOCT (52). All the MAGs obtained were aggregated and refined using the MetaWRAP-Bin_refinement module with the parameters, –c 70, –x 5, which indicated only MAGs with completion ≥ 70% and contamination ≤ 5% can be considered as target microbial genomes. Quality-controlled reads data were reassembled with the MetaWRAP-Reassemble_bins module to improve the N50, completeness, and contamination rate of the resulting MAGs set.

### Calculation of the MAGs abundance.

To estimate the relative abundance per microbial genome, we followed the protocol described by Zhou et al. (15). Minimap2 (v2.17-r941) mapped short paired-end quality-controlled reads per sample against reference genomic sequences of MAGs with the parameters “-ax sr” to generate base-level alignment information that was stored in sequence alignment map (SAM, file format) files (53). All SAM files were converted to compressed binary BAM files by SAMTools (v1.9) (54). CoverM (v0.6.0, https://github.com/wwood/CoverM) genome model was employed to calculate the relative abundance of each MAG based on the above-mentioned input BAM files and reference genomic FASTA files with the options “-m relative_abundance -x fa –min-read-aligned-percent 0.75 –min-read-percent-identity 0.95 –min-covered-fraction 0.”

### Taxonomic classification and phylogenetic identification.

Taxonomic annotation of MAGs was performed by using an open-source software Genome Taxonomy Database Toolkit (GTDB-Tk, v1.3.0) (55) and reference database GTDB release 95 (56). Gene function prediction of MAGs was executed using the MetaWRAP-Annotate_bins module with Prokka (57) prior to phylogenetic analysis. Phylogenetic tree was constructed using PhyloPhlAn (v3.0.58) (58) with the parameters, “-d phylophlan –diversity high –fast -t a -f supermatrix_aa.cfg,” and visualized with R package ggtree (v2.0.4) (59).

### Functional annotation.

The resulting predicted proteins of MAGs were annotated for CAZyme, nitrogen metabolism, and ARGs profiles. CAZyme genes were aligned to CAZy database (60) by using the HMMs of dbCAN sever with an E-value threshold of < 1e-18 and a minimum coverage of 35% (61). Nitrogen metabolic annotations were performed by KofamKOALA based on KEGG database with the options “-E 1e-5” on the local server (62). ARG-like proteins in MAGs were identified by a web server ARGs-OAP (v2.0) with function module SARGFAM based on an integrated SARG database (63). In brief, the genomic amino acid sequences were uploaded to the online program for ARGs annotation with default options “E-value of 1e-10, Similarity of 80% and alignment length of 70%.”

### Data analysis and visualization.

Statistical analyses and result visualization were performed using R Version 3.6.1 (64). Taxonomy classification and abundance were shown as Sankey diagram with R package “networkD3” (65). Heatmaps about CAZymes and ARGs were plotted with “pheatmap” package (66). Alpha- and beta-diversity of the snail’s microbiome between the groups was calculated with the “vegan” (67) and visualized with “ggplot2” package (68). Bray-Curtis distance was analyzed based on permutational multivariate analysis of variance (PERMANOVA), and the dissimilarities between and within groups were computed with analysis of similarities (ANOSIM). Discriminative genera between the two diets were identified using LEfSe in the Galaxy server (https://huttenhower.sph.harvard.edu/galaxy/) (69). The significance of differences between groups was assessed by using Wilcoxon test, and *P ≤ *0.05 was considered significant.

### Data availability.

Data sets supporting the conclusions of this study are available in the NCBI Sequence Read Archive (SRA) repository under accession No. PRJNA764360.
