# Supplementary material for: Metagenome-Assembled Genomes Reveal Mechanisms of Carbohydrate and Nitrogen Metabolism of Schistosomiasis-Transmitting Vector Biomphalaria Glabrata
Source: Microbiol Spectr. 2022 Mar 7;10(2):e01843-21. doi: 10.1128/spectrum.01843-21 (PMC9045156; doi:10.1128/spectrum.01843-21)
Supplement: SUPPLEMENTAL FILE 1 — Supplemental material. Download SPECTRUM01843-21_Supp_1_seq11.pdf, PDF file, 0.4 MB [file spectrum01843-21_supp_1_seq11.pdf]

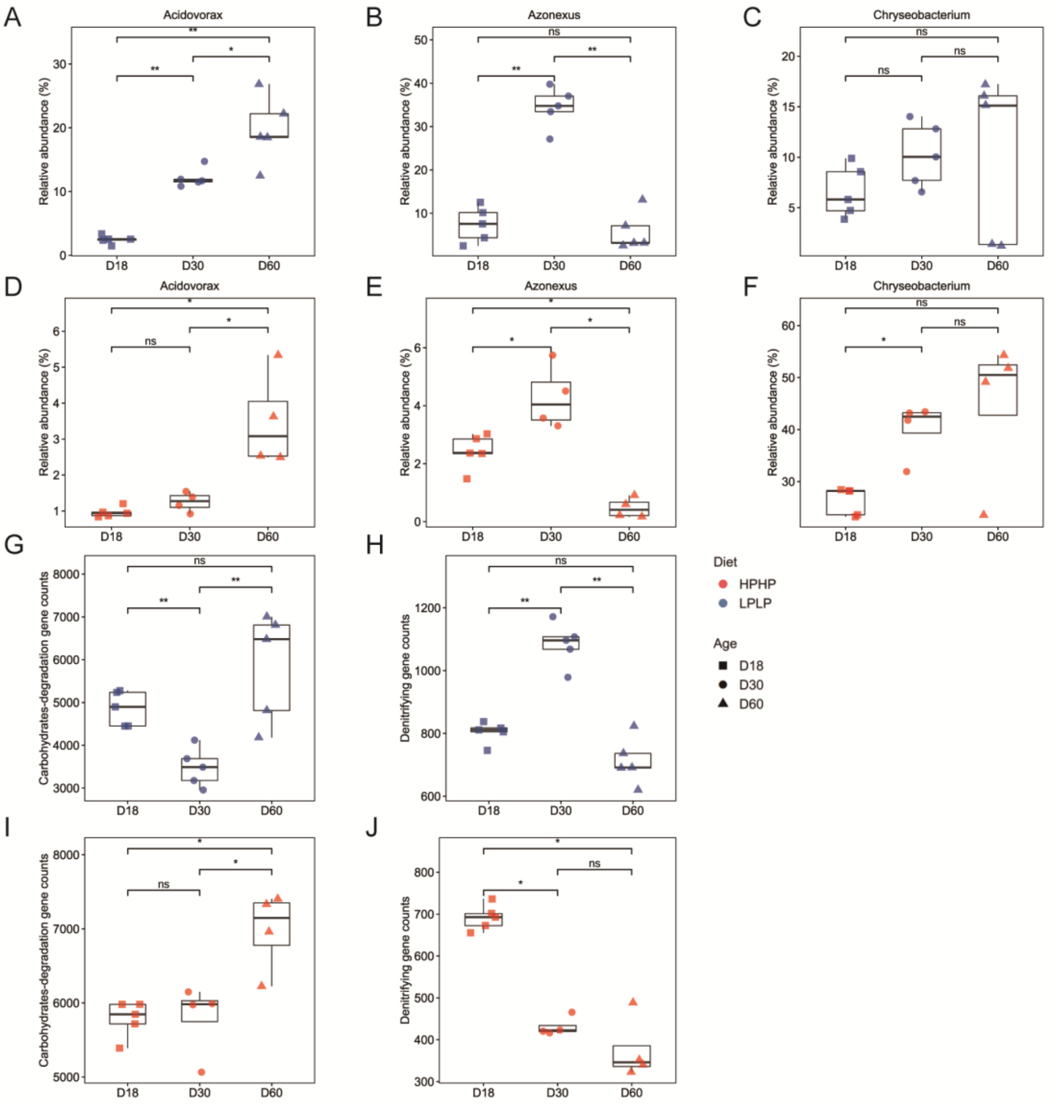

FIG. S1 Effect of age on *B. glabrata* gut bacterial community and function. (A–F) Change trends of three specific bacteria in the two diet groups over time. (G–J) Change trends of carbohydrate-degrading and denitrifying genes counts in the two diet groups over time. Statistical analysis was performed using Wilcoxon test.
